# Determining voluntary activation in synergistic muscles: a novel mechanomyographic approach

**DOI:** 10.1007/s00421-022-04966-8

**Published:** 2022-05-24

**Authors:** Emiliano Cè, Giuseppe Coratella, Christian Doria, Marta Borrelli, Susanna Rampichini, Eloisa Limonta, Stefano Longo, Fabio Esposito

**Affiliations:** 1 Department of Biomedical Sciences for Health (SCIBIS), Università Degli Studi Di Milano, University of Milan, Via Colombo 71, 20133 Milan, Italy; 2 IRCSS Galeazzi Orthopedic Institute, Via Riccardo Galeazzi, 4, 20161 Milan, Italy

**Keywords:** Interpolated twitch technique, Maximum voluntary contraction, Exercise-induced fatigability, Knee extensors, Potentiated force, Quadriceps

## Abstract

**Purpose:**

Drawing on correlations between the mechanomyographic (MMG) and the force signal, we devised a novel approach based on MMG signal analysis to detect voluntary activation (VA) of the synergistic superficial heads of the quadriceps muscle. We hypothesized that, after a fatiguing exercise, the changes in the evoked MMG signal of each quadriceps head would correlate with the changes in the level of VA in the whole quadriceps.

**Methods:**

Twenty-five men underwent a unilateral single-leg quadriceps exercise to failure. Before and after exercise, VA was assessed by interpolated-twitch-technique via nerve stimulation during and after maximum voluntary contraction (MVC). The force and MMG signal were recorded from *vastus lateralis*, *vastus medialis*, and *rectus femoris*. The MMG peak-to-peak was calculated and the voluntary activation index (VA_MMG_), defined as the superimposed/potentiated MMG peak-to-peak ratio, was determined from the MMG signal for each head.

**Results:**

VA_MMG_ presented a very high intraclass correlation coefficient (0.981–0.998) and sensitivity (MDC_95%_: 0.42–6.97%). MVC and VA were decreased after exercise in both the exercising [MVC:−17(5)%, ES −0.92; VA: −7(3)%, ES −1.90] and the contralateral limb [MVC: −9(4)%, ES −0.48; VA: −4(1)%, ES −1.51]. VA_MMG_ was decreased in both the exercising [~ −9(6)%, ES −1.77] and contralateral limb [~ −3(2)%, ES −0.57], with a greater decrease in VA_MMG_ noted only in the *vastus medialis* of the exercising limb. Moderate-to-very high correlations were found between VA_MMG_ and VA (*R-*range*:* 0.503–0.886) before and after exercise.

**Conclusion:**

VA_MMG_ may be implemented to assess VA and provide further information when multiple synergistic muscle heads are involved in fatiguing exercises.

**Supplementary Information:**

The online version contains supplementary material available at 10.1007/s00421-022-04966-8.

## Introduction

Voluntary activation (VA) of a skeletal muscle reflects the ability of the motor cortex system and several mechanisms downstream of the motor cortex system (i.e., the corticospinal tract and spinal/motoneuronal excitability) to activate a muscle by increasing the descending drive during voluntary contraction (Rekling et al. 2000; Gandevia 2001; de Haan et al. 2009). VA is typically assessed by the interpolated twitch technique, which involves an electrically evoked contraction delivered during maximum voluntary contraction (MVC), i.e., the peripheral nerve is stimulated during the MVC and the muscle fibers not activated by voluntary effort can be recruited (Merton 1954; Belanger and McComas 1981). Consequently, if a given motor unit is not firing fast enough to produce its maximal force, the superimposed action potential evokes a twitch-like increase in force from the whole muscle (Merton 1954; Belanger and McComas 1981). After the superimposed stimulation, a second twitch is evoked with the muscle at rest (potentiated twitch). The ratio between the superimposed and the potentiated twitch can then be calculated to define the level of VA (Merton 1954; Belanger and McComas 1981). In short, the more motor units recruited and the faster they fire during an MVC, the smaller the superimposed twitch amplitude, and the greater the VA (Merton 1954; Belanger and McComas 1981).

Although the interpolated twitch technique can give a valid estimation of the VA, it presents several methodological issues; for example, the compliance of the tendons and the myograph and/or the coupling to the myograph during contraction (Taylor 2009). Moreover, the inability to detect possible interference from the antagonist muscles (Allen et al. 1998), its sensitivity in assessing the post-activation potentiation mechanisms that could limit the comparison among different populations (e.g., children vs adults) (Dotan et al. 2021), and unfeasibility to detect possible differences between synergistic muscles in a given task should be also taken in consideration (Behm et al. 2002). This last point is particularly important, since most movements require several muscles acting in synergy, and the interpolated twitch technique cannot distinguish the VA of each individual muscle.

The differences in VA between synergistic muscles could be detected by combining the interpolated twitch technique with mechanomyography (MMG). MMG is a non-invasive approach that records and quantifies the low-frequency transverse oscillations propagating from the active muscle fibers to the skin surface during voluntary or evoked contraction by means of an accelerometer positioned on the muscle belly surface (Orizio 1993; Orizio et al. 2003; Cè et al. 2020b). The MMG signal can be recorded simultaneously from each individual superficial synergistic muscle so to differentiate the information simultaneously. Therefore, while the interpolated twitch provides information about the gross voluntary activation of the whole muscle group innervated by the same stimulated nerve, MMG can be used to distinguish the local mechanical response of each muscle.

Two phases of the MMG signal can be identified during the interpolated twitch technique procedure. In the first phase, the gross lateral movement of the contracting fibers at the beginning of the voluntary contraction (MMG peak-to peak during MVC, MMGp-p_MVC_) is generated by the shortening of the contractile elements before the slack of the elastic-connective tissue has been fully taken up and the force transmitted to the tendon insertion point (Orizio 1993). This phase is associated with the whole muscle activation: the greater the muscle activation, the greater its amplitude (Gobbo et al. 2006). Since the pioneering use of the MMG, it was hypothesized that the amplitude of this phase could correlate with the intensity of the active state, which in turn is correlated with the amount of Ca^2+^ released from the sarcoplasmic reticulum (Takamori et al. 1971). In the second phase, from the analysis of the vibrations at the resonance frequency of the muscle propagating toward the muscle surface and the skin, two variables can be calculated: the root mean square (RMS), which indirectly reflects the number of active motor units and the mechanical characteristics of the contractile and viscoelastic components (Orizio et al. 2003; Longo et al. 2014; Cè et al. 2015), and the mean frequency (MF), which indirectly mirrors the mean firing rate of the recruited motor unit pool (Orizio et al. 2003; Cè et al. 2015).

When the nerve stimulation is elicited using the interpolated twitch technique (superimposed or potentiated twitch), the MMG peak-to-peak recorded from each muscle can provide further information about their relative VA. The amplitude of the superimposed MMG peak-to-peak (MMGp-p_SUP_) is indeed correlated with the extent and the second derivative of the rate of force development of the superimposed twitch, suggesting that MMGp-p_SUP_ may reflect the excitation–contraction coupling of residual fibers not elicited by the voluntary output (Ohta et al. 2007). Differently, the single twitch MMG peak-to-peak at rest (MMGp-p_POT_) is reported to be more affected by the peripheral mechanisms of the muscle activation, such as excitation–contraction coupling (Orizio 1993; Orizio et al. 2003; Cè et al. 2020b). The correlations between the MMGp-p_POT_ amplitude and the potentiated twitch were reported in a previous study (Gobbo et al. 2006). Moreover, the correlations between the MMGp-p_SUP_ and the MMGp-p_POT_ amplitude with the superimposed and potentiated twitch amplitude indicate that the two MMG signal variables can be used to simultaneously determine the level of VA for each superficial single muscle head (Gobbo et al. 2006; Ohta et al. 2007, 2009). As for the force signal, a new variable can be calculated from the ratio between the MMGp-p_SUP_ and the MMGp-p_POT_ to identify the level of VA of the synergistic muscles, i.e., the VA recorded by the MMG signal (VA_MMG_).

The exercise-induced fatigability is one of the several factors that affect VA (Gandevia 2001). More specifically, the exercise-induced changes in VA during the knee extension have been so far assessed and identified for the quadriceps muscle as a whole (Gandevia 2001); however, the effect on VA of the single synergistic muscles performing the knee extension still needs to be investigated. A previous study reported morphological and histochemical differences in quadriceps muscle heads, with different type-I/type-II muscle fibers observed between the *Vastus lateralis* (*VL*) and the *Vastus medialis* (*VM*) (Travnik et al. 1995). Another study reported a trend for greater glucose uptake in the *VM* compared to the *VL* after a single-leg knee-extension exercise to exhaustion (Kalliokoski et al. 2011). Such differences may underlie the different effects of a fatiguing exercise on each single muscle head, resulting in different levels of reduction in VA between the quadriceps muscles. Finally, each muscle head could be differently regulated by the central nervous system (e.g., differences in corticomotoneuronal projections) during fatiguing exercise. With these points in mind, we wanted to: (i) determine exercise-induced changes in VA_MMG_ for each superficial head of the quadriceps muscle and (ii) test the relationship between the VA_MMG_ for each superficial head of the quadriceps muscle and the VA of the whole quadriceps muscle. For this purpose, we combined the interpolated twitch technique on a skeletal muscle with several heads (i.e., the quadriceps muscle) with an analysis of the MMG signal recorded from the *VL*, *VM,* and the *rectus femoris (RF)* to test the hypothesis.

## Methods

### Participants and ethical approval

Preliminary values were calculated in a subsample of ten participants from the correlation between VA and VA_MMG_ using a two-tailed bivariate correlation model (correlation *ρ *H1 = 0.866, correlation *ρ *H0 = 0.600, *α* = 0.05, 1-*β* err = 0.80); the required sample size was 23 participants (G-Power 3.1, Düsseldorf, Germany). On this basis, 25 healthy men [age 25 (1) yrs; body mass 77 (2) kg; stature 1.81 (0.02) m; mean (standard deviation)] took part in the study. Inclusion criteria were: no orthopedic and/or neurological disorders; no lower limb muscular or joint injury in the previous 6 months; and no involvement in an endurance exercise routine in the previous 6 months. The study was approved by the local University Ethics Committee (*CE* 27/17) and performed following the principles of the latest version of the Declaration of Helsinki. Written, informed consent was obtained from participants after a full explanation of the study purpose and the experimental design. Participants were informed that they could withdraw from the study at any time.

### Study design

**Fig. 1:**
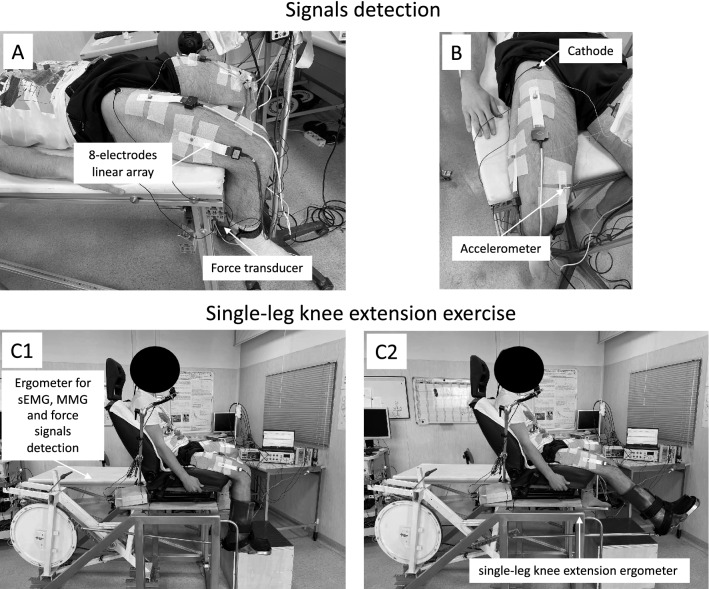
Experimental setup. Panel A and B: Mechanomyographic (*MMG*), surface electromyographic (*sEMG*), and force signal detection. Panel C1 and C2: the single-leg knee-extension ergometer used for the fatiguing exercise. The exercise was performed while sitting on an adjustable chair. Both knees were flexed at 90°, with the ankle of the exercising limb connected to a bicycle ergometer pedal arm by a rigid bar. The concentric phase occurred actively from 90° to the knees full extension, while the eccentric phase was driven passively by the flywheel inertia. The mechanical brake applied to the ergometer and the pedal frequency were measured to determine the mechanical power output. The mechanical friction, i.e., the force applied to each revolution, was measured by a force transducer, while the pedal frequency was determined by a magnetic transducer integrated in the cycle ergometer

For this cross-sectional study, the participants visited the laboratory four times. On the test days, they came after fasting overnight, having abstained from caffeine intake for at least 12 h and from heavy exercise for at least 48 h. During the first session, they were familiarized with the experimental set-up (single-leg knee-extension exercise), the MVC, and the interpolated twitch technique procedures. Identification points were mapped on the skin (moles, scars, and angiomas) and the position of stimulation and surface electromyography (sEMG) electrodes and accelerometers was drawn on transparency sheets for accurate repositioning of the electrodes in the same area (Cè et al. 2020a). During the second session, the MVC and the interpolated twitch technique procedures were repeated for testing reliability. The maximum work rate on a dynamic knee-extension ergometer was determined as described elsewhere (Laginestra et al. 2021). Briefly, we used a square-wave test starting with a 3 min workload of 15 W followed by increments of + 5 W interspersed by 5 min of recovery in between. The load of the last completed step was taken as the maximum work rate. During the third session, a single-leg knee-extension time-to-exhaustion trial at 85% of maximum work rate was performed. During the fourth session, the participants rested for a time equivalent to the time-to-exhaustion trial (Fig. 1). To ensure consistency, the third and four sessions were not randomized. During both sessions, the MVC of the knee extensor muscles and the stimulated twitch (elicited by 100-Hz doublets) were administered before (PRE) and within 2 min after (POST) the exercise or an equivalent time at rest. This interval was needed to let the participants move from the dynamic knee-extension ergometer to the ergometer used for the interpolated twitch technique (see Fig. 1).

In compliance with the interpolated twitch technique procedure, the stimulated twitch was elicited during the force plateau phase of the MVC (superimposed force) and after the MVC (potentiated force) to calculate VA. The knee extensors force and VL, VM, and RF MMG signals were detected during MVC to identify VA_MMG_ for each muscle. All measurements were taken on the limb doing the single-leg knee-extension exercise (exercising limb) and on the contralateral non-exercising limb. The exercising and contralateral non-exercising limb was randomized during the third session; their classification was maintained during the fourth session in line with previous procedures (Cè et al. 2020a). All measurements were performed in a laboratory kept at constant room temperature [20 (2 °C)] and humidity [50% (3%)]. To minimize circadian changes in force and joint mobility, the tests were conducted at the same hour between 9:00 AM and 12.00 noon.

### Between-muscle crosstalk

**Fig. 2:**
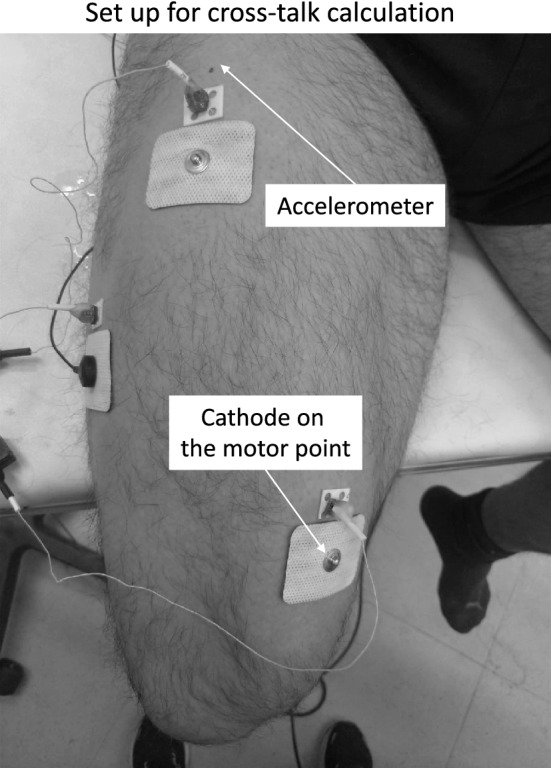
Experimental setup for the between-muscle crosstalk determination

Based on the sample size used in a previous study on the same investigated muscles (Beck et al. 2010), 12 participants were involved in a supplementary session to determine possible between-muscle crosstalk in the MMG signal during the electrical stimulation. During this session, the main motor point of one of the three synergistic muscles was supramaximally stimulated with a doublet using an inter-pulse duration of 10 ms. The participants laid supine, and the tested knee was flexed at 90° and firmly secured at the ankle with a Velcro® strap (Velcro Industries Inc., Willemstad, Netherlands Antilles), as shown in Fig. 2. After cleaning the skin with ethyl alcohol, the main motor point of *VL*, *VM*, and *RF* was localized by a pen electrode to position the cathode (45 × 35 mm rectangular electrode; Spes Medica, Battipaglia, SA, Italy) of the stimulator. A common anode (40 × 90 mm rectangular electrode; Spes Medica, Battipaglia, SA, Italy) was placed directly in contact with the other side of the thigh, as in a previous study (Gobbo et al. 2006). The MMGp-p_POT_ were measured on each muscle by monodirectional accelerometers [model ADXL103; Analog Devices, Norwood, MA, USA; device weight < 1.0 g; sensitivity 1000 mV·g^−1^; measure range (1.7 g)]. For each muscle, the stimulation amplitude generating the maximum MMGp-p_POT_ was detected with + 10 mA steps starting from 30 mA. The maximum amplitude was increased by + 10%. After 20 min of rest, the MMGp-p_POT_ generated by the stimulation on each synergistic muscle were recorded on each individual muscle separately. Each muscle was stimulated three times with 3 min of rest in between. The average MMGp-p_POT_ of each muscle was then calculated. The order of the muscle stimulation was randomized. The MMGp-p_POT_ elicited by a non-direct stimulation was normalized for the MMGp-p_POT_ elicited by a direct stimulation for each muscle.

### Measurements and data analysis

#### MVC

The MVC of the knee extensors was measured unilaterally on both limbs with the participants lying supine and the tested knee flexed at 90° and firmly secured at the ankle with a Velcro^®^ strap (Velcro Industries Inc., Willemstad, Netherlands Antilles) to a load cell (model SM-2000 N operating linearly between 0 and 2000 N; Interface, Crowthorne, UK) for force signal detection. The hips were kept extended at 0° and secured by a Velcro^®^ strap to the ergometer. After a standardized warm-up (10 2-s contractions at 50% MVC as determined during the familiarization session, followed by 10 2-s contractions at increasing intensity interspersed by 1 min each), two MVC attempts were performed at PRE and one MVC was performed at POST. If the MVC recorded at PRE differed by > 5%, further trials were performed until the difference decreased to < 5%. The participants were instructed to push as fast and as hard as they could for 4 s. Each MVC attempt was interspersed by at least 2 min of passive recovery. The force signal was driven to an A/D converter (model UM 150 Biopac; Biopac Systems Inc., Goleta, CA, USA), sampled at 1000 Hz, and stored on a personal computer. The maximum force recorded during the MVC was entered into data analysis.

#### VA and potentiated force

VA was calculated using the interpolated twitch technique. At baseline, the interpolated twitch technique was applied to elicit a superimposed doublet with an inter-pulse duration of 10 ms (100 Hz) (Cè et al. 2020a). During this procedure, the cathode (24 × 24 mm circle electrode; Spes Medica, Battipaglia, SA, Italy) was positioned over the femoral nerve under the iliopubic ligament, and the anode (40 × 90 mm rectangular electrode; Spes Medica) was placed 2 cm under the superior posterior iliac spine. The electrodes were connected to a high-voltage stimulator (Digitimer Stimulator model DS7AH, Hertfordshire, UK). The amperage of a square-wave pulse (1 ms, with an inter-pulse duration of 10 ms) was progressively increased until the maximum elicited force was achieved. Thereafter, the doublet was elicited during (superimposed force) and 5 s after each MVC (potentiated force). The force signal was analyzed with AcqKnowledge software ver. 4.4 (Biopac Systems). The VA was calculated as follows:

1 $$\mathrm{VA}=\left[100-\left(\frac{\mathrm{superimposed force}}{\mathrm{potentiated force}}\right)\times 100\right].$$

#### MMG signal assessment and analysis

**Fig. 3:**
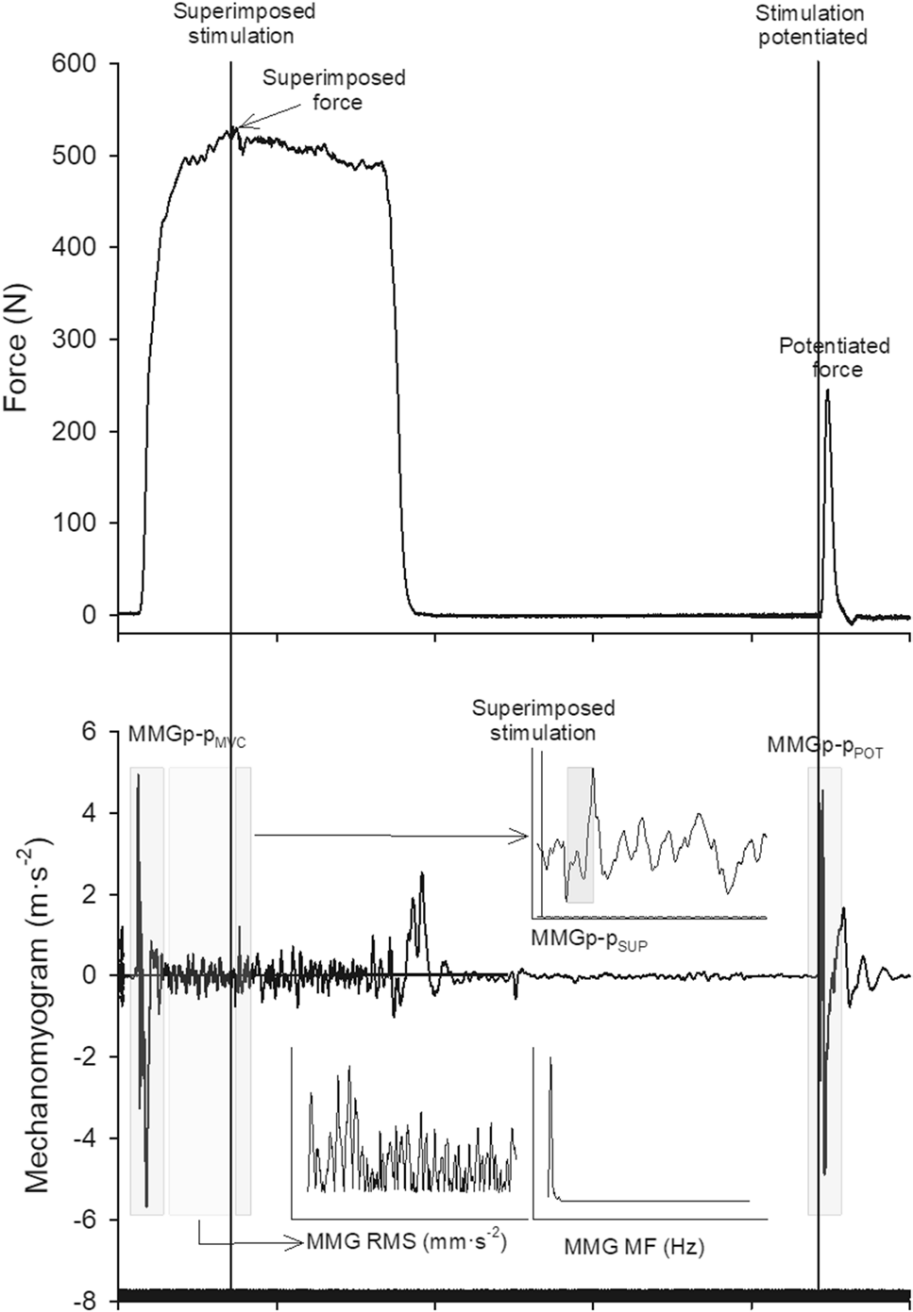
The mechanomyographic signal during the interpolated twitch technique. *MMGp-p*_*MVC*_ mechanomyographic signal peak-to-peak during the on phase of maximum voluntary contraction, *MMGp-p*_*SUP*_ mechanomyographic signal peak-to-peak elicited during superimposed stimulation, *MMGp-p*_*POT*_ mechanomyographic signal peak-to-peak elicited during potentiated stimulation, *RMS* denotes root mean square, *MF* mean frequency

The MMG signal was detected with three monodirectional accelerometers [model ADXL103; Analog Devices, Norwood, MA, USA; device weight < 1.0 g; sensitivity 1000 mV·g^−1^; measure range (1.7 g)] placed on the point of maximum vertical displacement during contraction (visually inspected) of *VL*, *VM*, and *RF* during the MVC and the potentiated doublet (Fig. 3). The signals were acquired at a sampling rate of 1000 Hz with an A/D converter (model UM 150 Biopac; Biopac Systems), filtered (filter-type IV-order Butterworth filter; bandwidth, 4–120 Hz) and stored on a personal computer for further analysis. The MMG signal analysis was performed with AcqKnowledge software ver. 4.4. The MMG signals were analyzed in the time and frequency domain within a 1-s time window detected in the middle of the MVC plateau preceding the superimposed stimulation. From this time window, the MMG, RMS, and MF (fast Fourier transform method) were calculated in consecutive 250-ms time windows and then averaged. The time delay from the stimulation to the onset of MMGp-p_POT_ was calculated and used to identify the MMGp-p_SUP_ [PRE: 8.5 (1.2) ms; POST: 9.3 (1.5) ms]. Similar to the calculation of VA and based on the correlations between the MMGp-p_SUP_ and the superimposed twitch (Ohta et al. 2007, 2009), and the MMGp-p_POT_ and the potentiated twitch (Gobbo et al. 2006), we identified the VA_MMG_ as follows:

2 $${\mathrm{VA}}_{\mathrm{MMG}}=\left[100-\left(\frac{{\mathrm{MMGp}-\mathrm{p}}_{\mathrm{SUP}}}{{\mathrm{MMGp}-\mathrm{p}}_{\mathrm{POT}}}\right)\times 100\right].$$

### sEMG signal assessment and analysis

The sEMG signal was detected during the MVC and the potentiated twitch in *VL*, *VM*, and *RF* by a linear array of eight electrodes (mod. ELSCH008; OtBioelettronica; probe 45 mm × 20 mm; electrode length 2 mm; interelectrode distance 10 mm) fixed to the skin by dual-adhesive foams (mod. AD004; OtBioelettronica) and filled with conductive gel (Cogel, Comedical, Trento, Italy). The skin area under the sEMG electrodes was cleaned with ethyl alcohol, abraded gently with fine sandpaper, and prepared with a conductive cream (Nuprep, Weaver and Co., Aurora, CO) to achieve an interelectrode impedance below 2000 Ω. For each muscle, the sEMG array was placed over the muscle belly along the direction of the muscle fibers, in accordance with the European recommendations for surface EMG (Hermens et al. 2000). The sEMG signal was acquired by a multichannel amplifier with a sampling rate of 2048 Hz (mod. EMG-USB; OtBioelettronica; input impedance: > 90 MΩ; CMRR: > 96 dB), amplified (gain × 1000), and filtered (filter type: IV-order Butterworth filter; bandwidth, 10–500 Hz) for further analysis. The sEMG analysis was performed by OtBiolab + software (OtBioelettronica). The signal was analyzed in a time domain within the same 1-s period as for the MMG signal; the sEMG RMS was calculated in consecutive 250 ms time windows and then averaged. The sEMG signal recorded during the potentiated twitch was exported as.csv file and converted in.acq file (AcqKnowledge 4.4; Biopac Systems). The software allowed the calculation of the maximum peak-to-peak of signal, which was considered as M-wave. The sEMG RMS/M-wave ratio was then calculated for each muscle.

### Fatiguing exercise

The fatiguing exercise (Fig. 1, Panel C1 and C2) was performed on a single-leg knee-extension ergometer with the participant seated on an adjustable chair with the ankle of the exercising limb attached by a rigid bar to a cycle ergometer (Monark, model 839E, Vansbro, Sweden) (Cè et al. 2021). During the exercise, the pedal rate (1 Hz) and the power output were displayed on a PC screen. The exercise was performed at 85% of maximum working rate until exhaustion (i.e., pedal rate < 1 Hz or a decrease in exercise power > 5% for 10 consecutive seconds) (Laginestra et al. 2021). In the control session, the participants lay supine and as relaxed as possible on a medical bed for a period equal to the fatiguing exercise duration.

## Statistical analysis

Statistical analysis was performed using a statistical software package (IBM SPSS Statistics 26, Armonk, NY). The Shapiro–Wilk test was used to check for normal distribution of the sampling. Greenhouse–Geisser correction was performed if the sphericity assumption was violated. The measurements taken during the first two sessions were utilized to calculate inter-session reliability and sensitivity. Reliability was calculated with a two-way random, consistency-type intraclass correlation coefficient (ICC). Cronbach’s α was classified as: very high (≥ 0.90); high (0.89 to 0.70); moderate (0.69 to 0.50) and the percentage standard error of the measurement (SEM%) was calculated. The minimum detectable change with a 95% confidence interval (MDC_95%_) defined sensitivity. PRE–POST differences in MVC, potentiated force, and VA after the fatiguing exercise and control session were evaluated in the exercising and contralateral non-exercising limb by three-way (time × exercise × limb) analysis of variance (ANOVA) for repeated measures. To calculate the between-muscle difference (*VL*, *VM*, *and RF*) in the MMG and sEMG variables, four-way (time × exercise × limb × muscle) ANOVA for repeated measures was performed. Further analysis of covariance (ANCOVA) was performed for force, MMG, and sEMG variables taking the baseline values as covariate. Multiple comparisons were adjusted with Bonferroni’s correction. The magnitude of interactions and single factors were calculated using partial eta squared (*η*_p_^2^). The magnitude of pairwise changes was determined by Cohen’s *d* effect size (ES). Cohen’s *d* was classified as *trivial* (0–0.19), *small* (0.20–0.59), *moderate* (0.60–1.19), *large* (1.20–1.99), and *very large* (≥ 2.00) (Hopkins et al. 2009). *η*^*2*^_P_ was classified as *small* (0.01–0.059), *medium* (0.06–0.139), and *large* (≥ 0.14). Pearson’s moment product test with bootstrap method correction checked for correlations between VA_MMG_ and VA and between VA_MMG_ and the sEMG variables. The magnitude of the correlations was classified as *trivial* for a coefficient (*R*) < 0.1, *small* for *R* between 0.11 and 0.30, *moderate* for *R* between 0.31 and 0.50, *high* for *R* between 0.51 and 0.70, *very high* for *R* between 0.71 and 0.90, *nearly perfect* for *R* between 0.91 and 0.99, and *perfect* for *R* = 1 (Hopkins et al. 2009). The determination coefficient (*R*^2^) was also calculated. Statistical significance was set with *P* value < 0.05. Unless otherwise stated, descriptive statistics are presented as mean (SD).

## Results

The single-leg knee-extension exercise was 478(132) s in duration. Table 1 presents the reliability (ICC and SEM%) and the sensitivity variables (MDC_95%_). The ICC ranged from 0.981 to 0.998 and the SEM% from 0.21% to 3.56%. MDC_95%_ ranged from 0.42% to 6.97%.

**Table 1:** Intersession reliability [ICC with its 95% confidence interval (CI_95%_) and SEM%] and sensitivity (MDC_95%_) for each dependent parameter. *ICC* denotes intraclass correlation coefficient, *SEM%* percentage standard error of measurement, *MDC*_*95%*_ minimum detectable change with a 95% confidence interval, *m* mean, *SD* standard deviation, *VA*_*MMG*_ voluntary activation calculated on the mechanomyographic signal, *MMG* mechanomyogram, *p-p*_*MVC*_ peak-to-peak during maximum isometric voluntary contraction, *p-p*_*SUP*_ peak-to-peak elicited during superimposed stimulation, *p-p*_*POT*,_ peak-to-peak elicited during potentiated stimulation, *VL Vastus lateralis*, *VM Vastus medialis*, *RF, rectus femoris*

		Trial 1 [m(SD)]	Trial 2 [m(SD)]	ICC (CI_95%_)	SEM%	MDC_95%_
VA_MMG_ (%)	*VL*	87.9 (4.1)	87.4 (4.3)	0.998 (0.997 – 0.999)	0.215	0.421
	*VM*	87.5 (3.8)	87.4 (3.4)	0.985 (0.970 – 0.990)	0.505	0.991
	*RF*	87.4 (4.2)	88.1 (4.4)	0.984 (0.978 – 0.986)	0.617	1.209
MMGp-p_MVC_ (mm·s^−2^)	*VL*	8.51(2.13)	8.22 (2.05)	0.985 (0.981 – 0.987)	3.062	6.002
	*VM*	8.64 (2.11)	8.18 (2.11)	0.987 (0.984 – 0.989)	2.857	5.600
	*RF*	8.61 (2.18)	8.10 (2.13)	0.981 (0.979 – 0.982)	3.555	6.968
MMGp-p_SUP_ (mm·s^−2^)	*VL*	0.95 (0.36)	0.95 (0.35)	0.998 (0.995 – 0.999)	1.691	3.314
	*VM*	0.99 (0.35)	0.99 (0.34)	0.994 (0.986 – 0,997)	2.698	5.288
	*RF*	0.98 (0.34)	0.97 (0.35)	0.996 (0.992 – 0.998)	2.223	4.356
MMGp-p_POT_ (mm·s^−2^)	*VL*	7.84(1.92)	7.84 (1.86)	0.992 (0.893 – 0.998)	2.156	4.227
	*VM*	7.94 (1.89)	7.93 (1.90)	0.994 (0.520 – 0.991)	1.853	3.633
	*RF*	7.91 (1.94)	7.90 (1.90)	0.996 (0.543 – 0.988)	1.539	3.017

### Between-muscle crosstalk

**Fig. 4:**
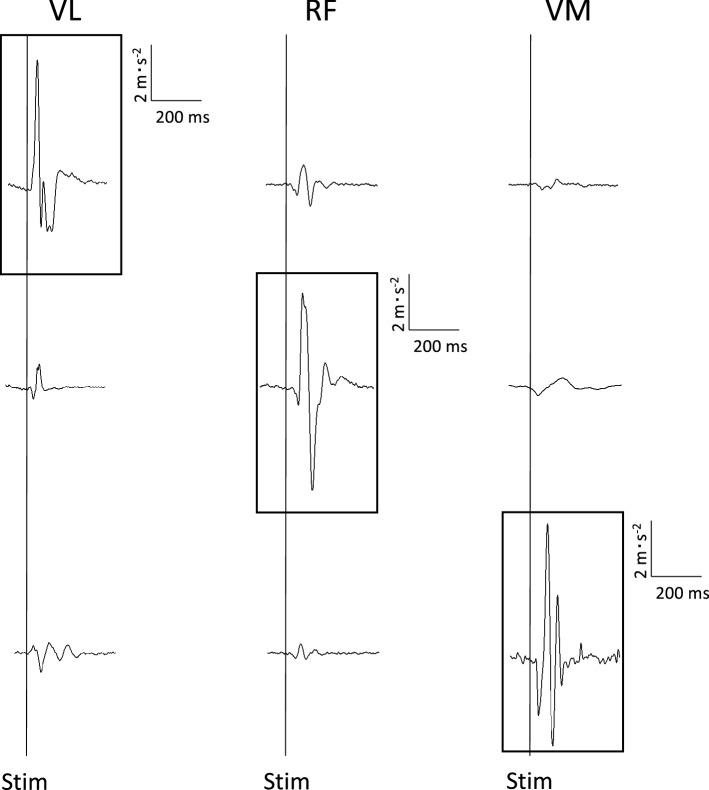
Between-muscle crosstalk. The potentiated mechanomyographic peak-to-peak signal of *Vastus lateralis* (*VL*), *vastus medialis* (*VM*), and *rectus femoris* (*RF*) is shown in response of specific (framed signal) and non-specific muscle stimulations

The MMG signal response of the other synergistic muscles during the stimulation of the main motor point of one muscle is provided in Fig. 4 from a representative participant. Table 2 reports the MMGp-p_POT_ elicited during the direct stimulation of one muscle and those from the muscle not directly stimulated. Crosstalk signals ranging from the 4(1)% to 11(2)% were found from the muscles not directly stimulated.

**Table 2:** Between-muscle crosstalk. *MMG* mechanomyogram, *p-p*_*POT*,_ peak-to-peak elicited during potentiated stimulation, *VL Vastus lateralis*, *VM Vastus medialis*, *RF Rectus femoris*

	Stimulated muscle	Crosstalk					
	MMGp-p_POT_ (m⋅s^−2^)	*VL* MMGp-p_POT_ (m⋅s^−2^)	Δ%	*VM* MMGp-p_POT_ (m⋅s^−2^)	Δ%	*RF* MMGp-p_POT_ (m⋅s^−2^)	Δ%
*VL*	7.5 (1.2)	–	–	0.4 (0.2)	6 (3)	0.8 (0.2)	11 (2)
*VM*	7.4 (1.0)	0.8 (0.1)	11 (2)	–	–	0.3 (0.1)	4 (1)
*RF*	7.5 (1.2)	0.8 (0.1)	11 (2)	0.7 (0.1)	10 (2)	–	–

### MVC

**Fig. 5:**
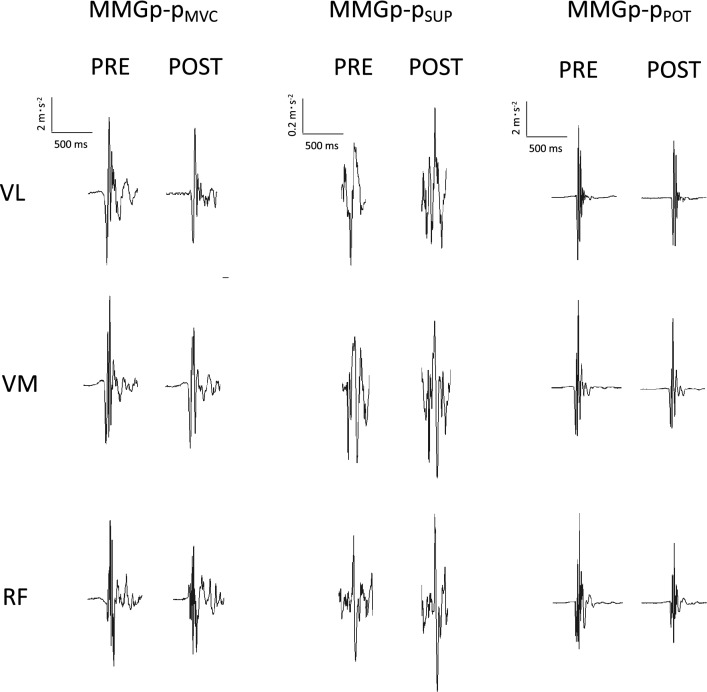
PRE–POST changes in: MMG peak-to peak during maximum voluntary contraction (*MMGp-p*_*MVC*_), superimposed (*MMGp-p*_*SUP*_), and potentiated twitch (*MMGp-p*_*POT*_) for a representative participant

The ANOVA disclosed a time × exercise × limb interaction for MVC (*F*_1_,_143_ = 20.81, *P* < 0.001, *η*^2^_P_ = 0.201). MVC (Fig. 5, Panel A) was reduced in both limbs after exercise [exercising limb −17(5)%, *P* < 0.001, ES = −0.92 (−1.20 to −0.63); contralateral non-exercising limb -9(4)%, *P* < 0.001, ES = −0.48 (−0.75 to −0.21)], with larger decreases in the exercising limb compared to the contralateral non-exercising limb [*P* < 0.001, ES = −0.42 (−0.70 to −0.15)]. No changes were observed in the control (*P* > 0.05).

### Potentiated force

The ANOVA disclosed a time × exercise × limb interaction for potentiated force (*F*_1,__143_ = 16.40, *P* < 0.001, *η*^*2*^_P_ = 0.146). A reduction in potentiated force (Fig. 5. Panel B) was observed after exercise only in the exercising limb [−18(8)%, *P* < 0.001, ES = −0.97 (−1.26 to −0.69)]. No changes were observed in the contralateral non-exercising limb or the control (*P* > 0.05).

### VA

The ANOVA disclosed a time × exercise × limb interaction for VA (*F*_1,__143_ = 17.37, *P* < 0.001, *η*^*2*^_P_ = 0.155). A decrease in VA (Fig. 5, panel C) was observed in both limbs after exercise [exercising limb: −7(3)%, *P* < 0.001, ES = −1.90 (−2.22 to −1.57); contralateral non-exercising limb: −4(1)%, *P* < 0.001, ES = −1.51 (−1.81 to −1.20)] with a greater decrease in the exercising limb compared to the contralateral non-exercising limb [*P* < 0.001, ES = −0.87 (−1.15 to −0.59)]. No differences were noted in the control (*P* > 0.05).

### VA_MMG_

**Fig. 6:**
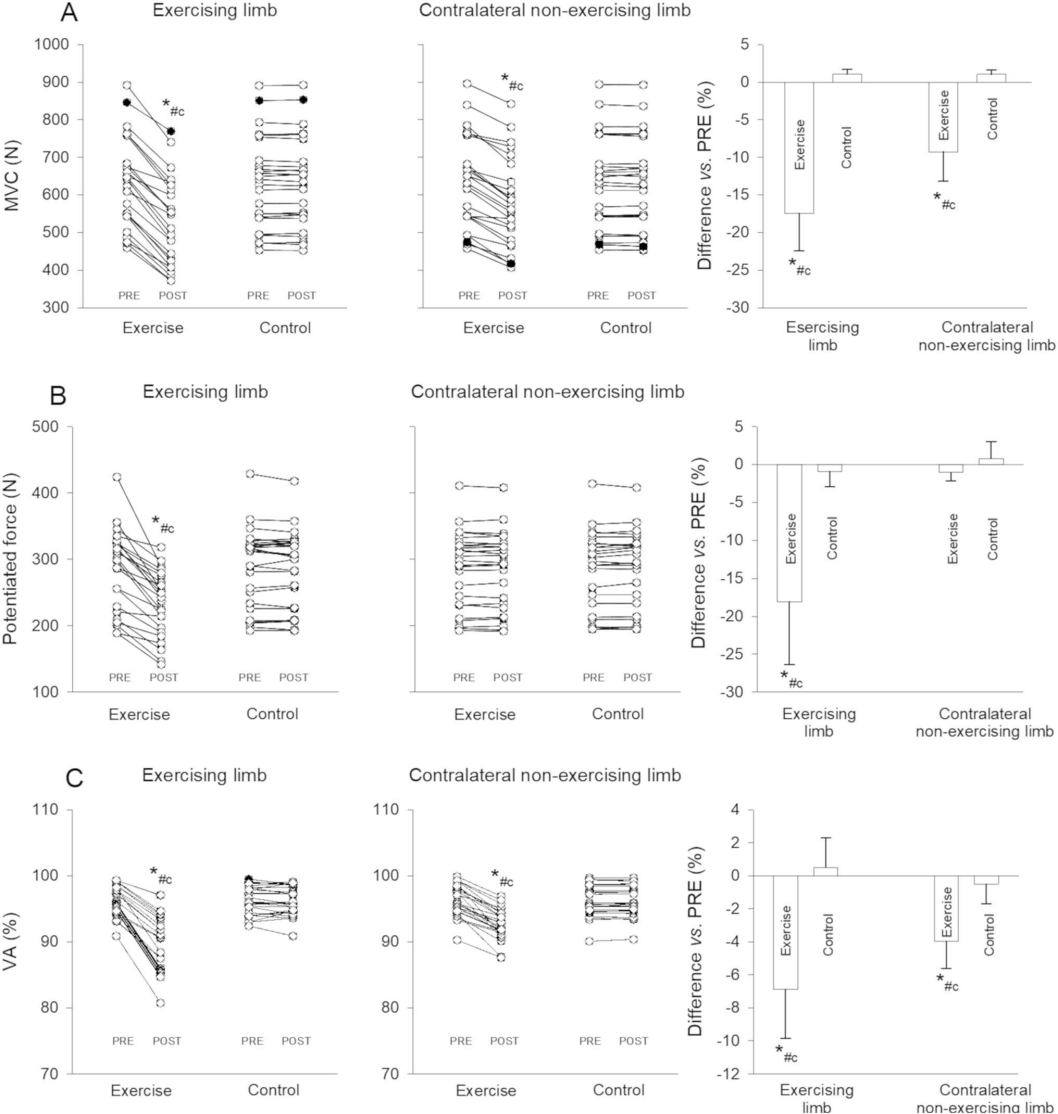
Individual data and percentage changes in maximum voluntary contraction (*MVC*, panel A), potentiated force (panel B), and voluntary activation from force signal analysis (*VA*, panel C) of the knee extensor after exercise or control in the exercising and the contralateral limb *P* < 0.05 post *vs*. PRE ^c^*P* < 0.05 *vs.* control ^#^*P* < 0.05 contralateral non-exercising *vs*. exercising limb

The ANOVA showed a time × exercise × limb × muscle interaction for VA_MMG_ (*F*_1,431_ = 18.00, *P* < 0.001, *η*^2^_P_ = 0.111). A difference in decrease in VA_MMG_ was noted between *VL*, *VM*, and *RF* of both limbs after exercise [exercising limb: ES-range, −2.15 – −1.00; contralateral non-exercising limb: ES range, −0.82 – −0.72], while no changes were observed in the control (*P* > 0.05) (Fig. 6). The decrease in VA_MMG_ of the *VM* in the exercising limb was greater than in *VL* [*P* < 0.001, ES = −0.35 (−0.62 to -0.08)] and *RF* [*P* < 0.001, ES = −0.96 (−1.24 to −0.68)]. Larger decreases in VA_MMG_ were found in the exercising limb compared to the contralateral non-exercising limb.

### MMG RMS

The ANOVA showed a time × exercise × limb × muscle interaction for MMG RMS (*F*_1,431_ = 3.90, *P* = 0.02, *η*^2^_P_ = 0.026). After exercise, MMG RMS was decreased in both the exercising and the contralateral non-exercising limb (*P* < 0.001 for all muscles), with larger decreases noted in the exercising limb compared to the contralateral non-exercising limb (ES-range = −1.73 to −1.00). A larger decrease in MMG RMS was found in the *VM* of the exercising limb than in *VL* [*P* < 0.001, ES = −1.13 (−1.22 to −0.84)] and *RF* [*P* < 0.001, ES = −1.00 (−1.28 to −0.71)]. No changes were observed in the control (*P* > 0.05).

### MMG MF

The ANOVA showed a time × exercise × limb × muscle interaction for MMG MF (*F*_1,431_ = 6.42, *P* = 0.002, *η*^2^_P_ = 0.043). A decrease in MMG MF was observed after exercise, with larger decreases noted in the exercising limb compared to the contralateral non-exercising limb [ES-range = −1.26 to −0.92]. No changes were observed in the control (*P* > 0.05). The decrease in MMG MF in the *VM* of the exercising limb was larger than in *VL* [*P* = 0.04, ES = −0.31 (−0.58 to −0.04)] and *RF* [*P* < 0.001, ES = −0.54 (−0.81 to −0.26)].

The Cohen’s *d* ES with 95% CI of the changes in MMG RMS and MF are shown in Table, Supplemental Digital Content.

### MMGp-p_MVC_

The ANOVA showed a time × exercise × limb × muscle interaction for MMGp-p_MVC_ (*F*_1,431_ = 5.34, *P* = 0.005, *η*^2^_P_ = 0.036). After exercise MMGp-p_MVC_ was decreased in both the exercising limb and the contralateral non-exercising limb (*P* < 0.001 for all muscles). No changes were observed in the control (*P* > 0.05). The decrease in MMGp-p_MVC_ was larger in the *VM* compared to *VL* [*P* < 0.001, ES = −1.18 (−1.47 to −0.89)] and *RF* [*P* < 0.001, ES = −1.16 (−1.45 to –0.87)].

### MMGp-p_SUP_

The ANOVA showed a time × exercise × limb × muscle interaction for MMGp-p_SUP_ (*F*_1,431_ = 6.76, *P* = 0.001, *η*^2^_P_ = 0.045). MMGp-p_SUP_ was increased after exercise in both the exercising limb and the contralateral non-exercising limb (*P* < 0.001 for all muscles). No changes were observed in the control (*P* > 0.05). The increase in MMGp-p_SUP_ was greater in the *VM* than in *VL* [*P* < 0.001, ES 0.85 (0.57 to 1.13)] and *RF* [*P* < 0.001, ES 0.48 (0.20 to 0.75)] of the exercising limb.

### MMGp-p_POT_

The ANOVA showed a time × exercise × limb × muscle interaction for MMGp-p_POT_ (*F*_1,431_ = 5.46, *P* = 0.005, *η*^2^_P_ = 0.037). MMGp-p_POT_ was decreased after exercise only in the exercising limb (*P* < 0.001 for all muscles), while it was unchanged in the contralateral non-exercising limb and the control (*P* > 0.05). The decrease in MMGp-p_POT_ was greater in the *VM* than in *VL* [*P* < 0.001, ES = −1.23 (−1.52 to −0.94)] and *RF* [*P* < 0.001, ES = −0.34 (−0.61 to −0.07)].

**Fig. 7:**
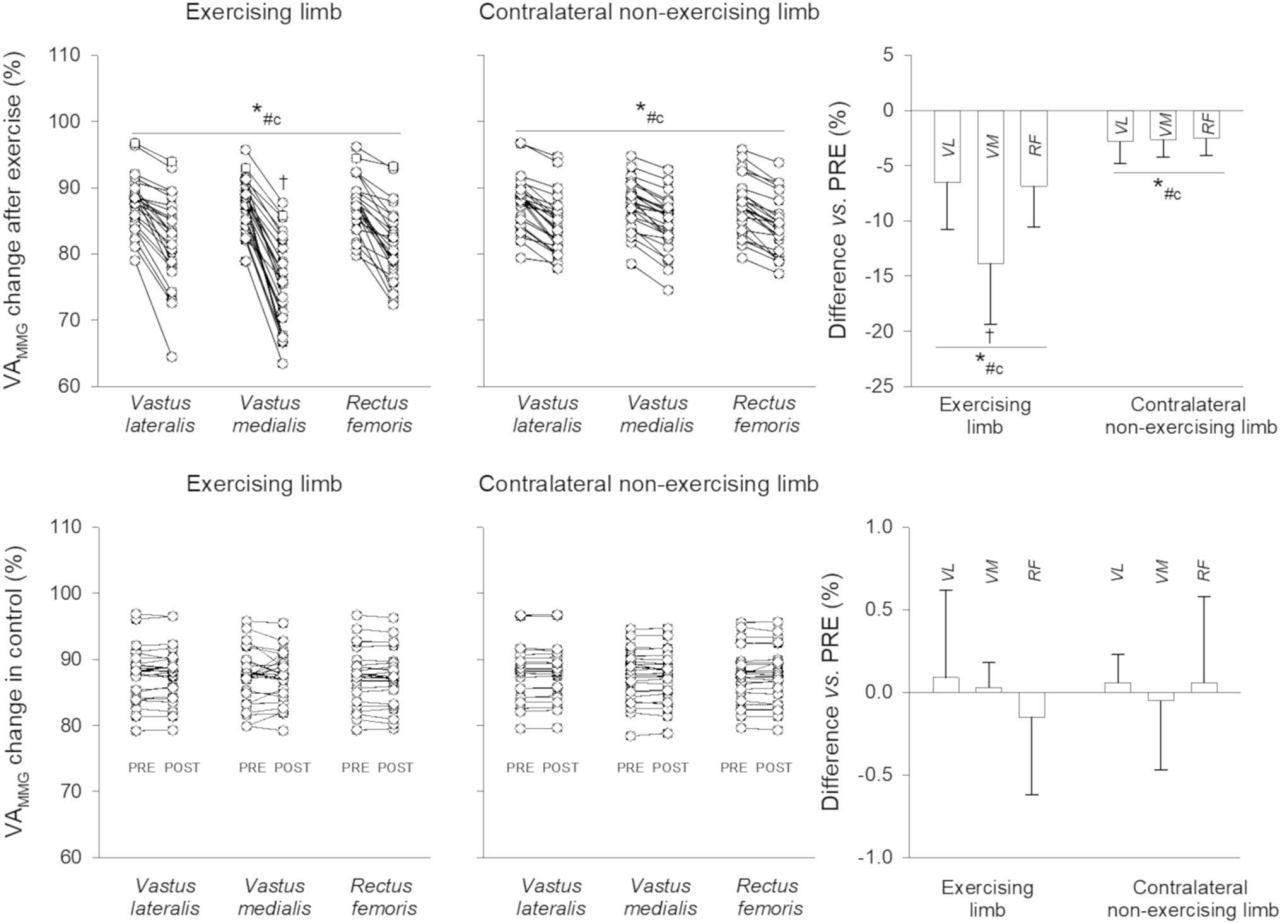
Individual data and percentage changes in voluntary activation from mechanomyographic signal analysis (VA_MMG_) after exercise (upper panels) or control (lower panels) in the exercising and the contralateral non-exercising limb. *VL, vastus lateralis*; *VM, vastus medialis*; *RF, rectus femoris *.^*^P < 0.05 post *vs*. PRE ^c^*P* < 0.05 *vs.* control ^#^*P* < 0.05 contralateral non-exercising *vs.* exercising limb ^†^*P* < 0.05 *Vastus medialis* vs. *Vastus lateralis* and *Rectus femoris*.

The PRE-POST changes in MMGp-p_MVC_, MMGp-p_SUP_, and MMGp-p_POT_ for a representative participant are shown in Fig. 7. Cohen’s *d* ES with 95% CI of the changes in MMGp-p_MVC_, MMGp-p_SUP_, and MMGp-p_POT_ are shown in Table, Supplemental Digital Content 2.

### sEMG RMS

The ANOVA showed a time × exercise × limb × muscle interaction for sEMG RMS (*F*_1,431_ = 4.08, *P* < 0.001, *η*^2^_P_ = 0.555). After exercise sEMG RMS was decreased in both the exercising limb and the contralateral non-exercising limb (*P* < 0.001 for all muscles), whereas no changes were observed in the control (*P* > 0.05). The decrease in sEMG RMS was larger in *VM* compared to *VL* [*P* < 0.001, ES = −0.95 (−1.53 to −0.36)] and *RF* [*P* < 0.001, ES = −0.82 (−1.40 to −0.24)].

### M-wave

The ANOVA did not show a time × exercise × limb × muscle interaction for M-wave. M-wave decreased only in the exercising limb by about 7(6)% with no between-muscle differences. No changes occurred in the contralateral non-exercising limb and in control.

### sEMG RMS/M-wave

The ANOVA did not show a time × exercise × limb × muscle interaction for sEMG RMS/M-wave. After exercise sEMG RMS/M-wave was decreased in both the exercising limb and the contralateral non-exercising limb (*P* < 0.001 for all muscles), whereas no changes were observed in the control (*P* > 0.05). The decrease in sEMG RMS/M- of the exercise limb wave was larger in *VM* compared to *VL* [*P* < 0.001, ES = −0.92 (−1.50 to −0.36)] and *RF* [*P* < 0.001, ES = −0.81 (−1.38 to −0.23)].

Cohen’s *d* ES with 95% CI of the changes in sEMG RMS, M-wave, and sEMG RMS/M-wave are shown in Table, Supplemental Digital Content 3.

### Correlations

**Fig. 8:**
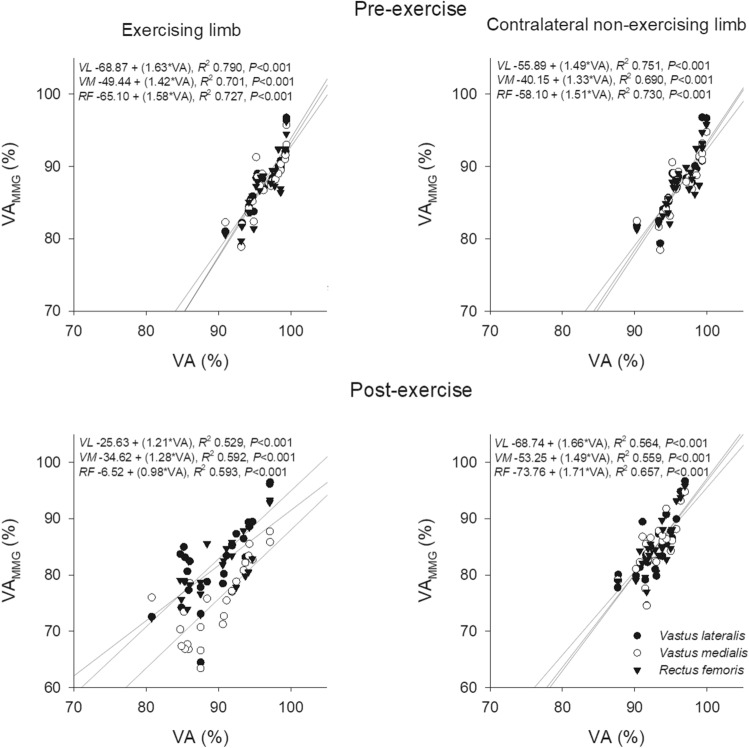
The correlations between voluntary activation determined from analysis of the mechanomyographic (*VA*_*MMG*_) and the force signal (*VA*) for the baseline (upper panels) and the post-exercise measurement (lower panels) in the exercising limb and the contralateral non-exercising limb

**Fig. 9:**
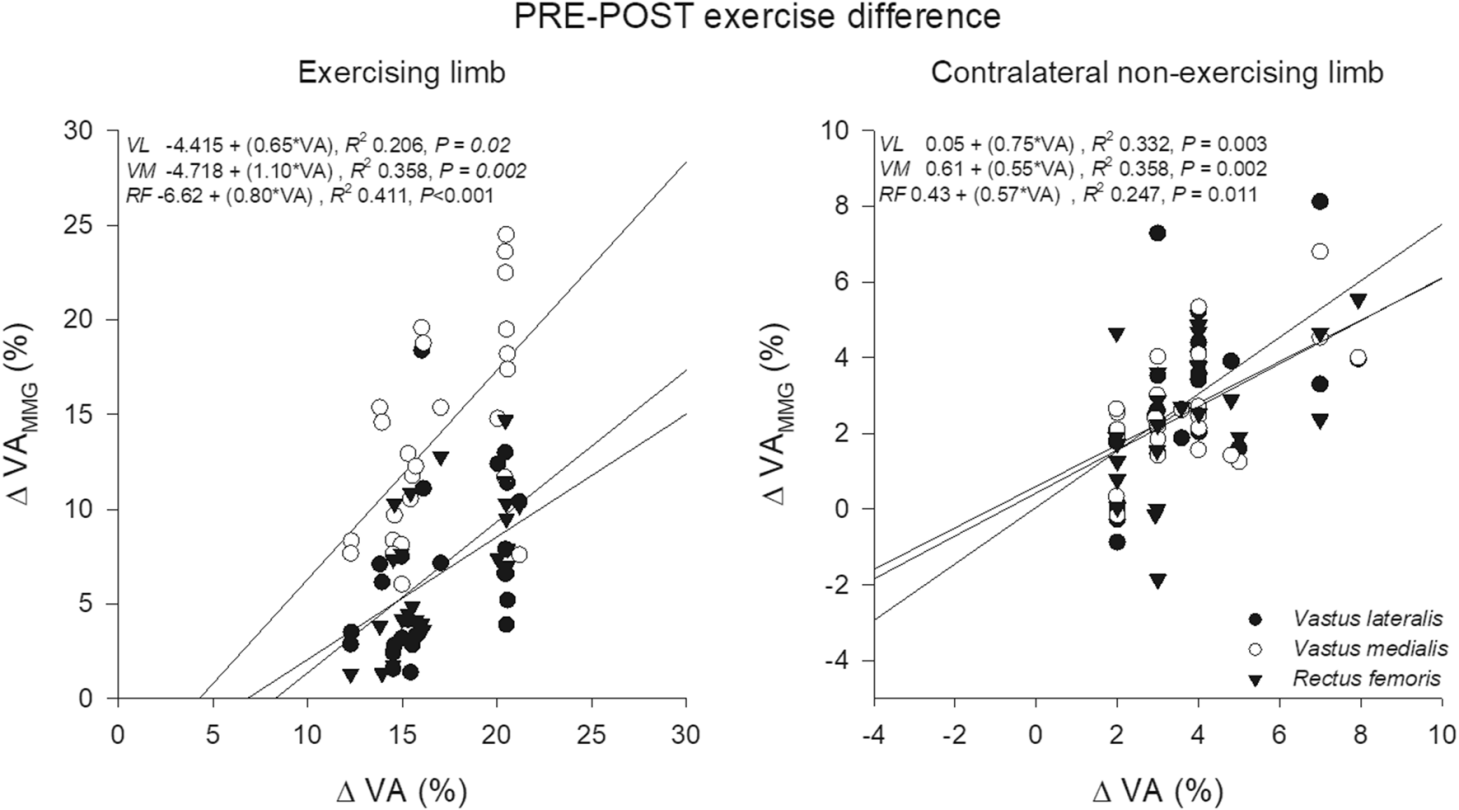
The correlations between voluntary activation determined from analysis of the mechanomyographic (*VA*_*MMG*_) and the force signal (*VA*) for PRE–POST changes in the exercising limb and the contralateral non-exercising limb

The correlations between VA_MMG_ and VA are shown in Fig. 8 (PRE or POST) and Fig. 9 (PRE–POST exercise changes). *Moderate*-to-*very high* positive correlations were observed for both measures and the exercise-induced changes in VA_MMG_ and VA before exercise (*P* < 0.05). On the contrary, no correlations were found between VA_MMG_ and the sEMG variables when considering raw data in PRE and POST, or when considering as PRE-POST exercise changes (PRE: *R-*range, −0.363 – 0.364; *R*^2^-range, 0.006 – 0.133; POST: *R*-range, −0.292 – 0.357; *R*^*2*^-range, 0.002 – 0.127; D% PRE-POST: *R-*range, −0.123 – 0.234; *R*^2^*-*range, 0.015 – 0.055*; P* > 0.05 for all comparisons).

Positive *high*-to-*very high* correlations were found between superimposed twitch and MMGp-p_SUP_ (PRE: *R-*range, 0.721 – 0.790; *R*^2^-range, 0.520 – 0.624; POST: *R*-range, 0.674 – 0.815; *R*^2^-range, 0.455 – 0.667; *P* < 0.001 for all comparisons) and between potentiated twitch and MMGp-p_POT_ (PRE: *R*-range, 0.616 – 0.790; *R*^2^-range, 0.379 – 0.462; POST: *R*-range, 0.633 – 0.815, *R*^2^-range, 0.401 – 0.667; *P* < 0.001 for all comparisons). Positive correlations were also found between the percentage of exercise-induced changes in superimposed twitch and MMGp-p_SUP_ in the *VL* [*R* = 0.453 (*moderate*), *R*^2^ = 0.205, *P* = 0.023] and *RF* of the exercising limb [*R* = 0.584 (*high*), *R*^2^ = 0.342, *P* = 0.002].

## Discussion

The present study was designed to test our hypothesis that the changes in VA_MMG_ would differ in each superficial head of the quadriceps following a fatiguing exercise, and that VA_MMG_ performed in *VL*, *VM* and *RF* could be used to gain information on VA assessed by the interpolated twitch technique performed on the quadriceps. As expected, a decrease in the VA of the quadriceps of the exercising limb was noted after the fatiguing exercise. Concomitantly, VA_MMG_ decreased in all examined quadriceps heads, with *VM* demonstrating a larger decrease compared to both *VL* and *RF*. VA_MMG_ correlated positively with VA in both the exercising and the contralateral non-exercising limb. Finally, VA_MMG_, MMGp-p_SUP_ and MMGp-p_POT_ presented an overall high inter-session reliability and adequate MDC_95%_.

### Preliminary considerations

In line with the previous studies, a decrease in MVC, VA, potentiated force, and sEMG RMS was observed after single-leg knee-extension exercise in both the exercising and non-exercising limb (Gandevia 2001; Rattey et al. 2006; Martin et al. 2008; Doix et al. 2013; Elmer et al. 2013; Kennedy et al. 2015; Jalal Aboodarda et al. 2019; Whitten et al. 2021). The variations in muscle metabolites induced in the exercising limb by the fatiguing exercise, i.e., an increase in intracellular inorganic-P, K^+^ (Johnson et al. 2014), H^+^ (Bangsbo et al. 1996; Johnson et al. 2014), blood lactate concentration (Bangsbo et al. 1996; Halperin et al. 2014; Johnson et al. 2014), and a decrease in Ca^2+^ availability (Fitts 1994), trigger an increase in group III/IV muscle afferent feedback at both supraspinal and spinal level. This feedback leads to a reduction in motor neuron excitability and firing rate, and results in a motor drive inhibition (Martin et al. 2008; Kennedy et al. 2015) toward the exercising limb, and in the contralateral non-exercising limb via neural pathways implicated in the crossover effect phenomenon (Rattey et al. 2006; Doix et al. 2013; Laginestra et al. 2021; Whitten et al. 2021). In addition to the central mechanisms, the reduction in M-wave and potentiated force in the exercising limb suggests a possible impairment in sarcolemma conduction properties and of cross-bridge cycle efficiency (Hultman et al. 1985; Cady et al. 1989; Fitts 1994; Bangsbo et al. 1996; Kent-Braun 1999; Halperin et al. 2014; Johnson et al. 2014).

### Effect of the fatiguing exercise on VA_MMG_

In the exercising limb, VA_MMG_ was decreased in all muscle heads but more so in the *VM* muscle. The changes were accompanied by a decrease in MMGp-p_MVC_, MMGp-p_POT_, MMG RMS, MMG MF, sEMG RMS, and sEMG RMS/M-wave in all muscle heads, with a greater decrease observed in the *VM* muscle. These decrements were also accompanied by reductions in M-wave amplitude occurring with a similar extent in all the three muscle heads. MMGp-p_MVC_, MMG RMS, and sEMG RMS are the gross sum of central and peripheral mechanisms underlying exercise-induced fatigability (Orizio et al. 2003). To explore these aspects, we added MMG MF, which reflects the central mechanisms of exercise-induced fatigability, since it indirectly monitors the firing rate of activation of the motor neurons responsible for muscle contraction (Orizio 1993; Cè et al. 2015, 2020b), and the M-wave and MMGp-p_POT_, which are more influenced by peripheral mechanisms related to exercise-induced fatigability (Orizio et al. 2003). The changes in MMG are consistent with the changes observed using the interpolated twitch technique. However, while the interpolated twitch technique cannot provide any further information about the synergistic muscle heads involved in a fatiguing task, the MMG can distinguish it. Furthermore, greater fatigue was noted in the *VM* muscle than the other superficial muscle heads.

The present outcomes seem to suggest that the larger decrease in VA_MMG_ in the *VM* could be induced by both central and peripheral fatigue mechanisms. The between-muscle difference in sEMG RMS/M-wave and MMG MF (more related to central mechanisms), together with the MMGp-p_POT_, seems to suggest a role is played by both the central and peripheral mechanisms linked to fatigue. Using a similar single-leg knee-extension exercise, a previous study found a trend of *VM* exhibiting larger glucose uptake than *VL* or *RF* muscle, as detected by positron emission tomography (Kalliokoski et al. 2011). This occurrence could have induced two different possible mechanisms, one more “central” and the other more “peripheral” in nature: (i) the greater metabolic involvement of the *VM* might have generated greater afferent feedback from group III/IV fibers (which are sensitive to the changes in the metabolic milieu). This in turn may have induced a greater inhibition of the descending drive in *VM* compared to *VL* and *RF*; (ii) the greater metabolic involvement of the *VM* muscle during the fatiguing exercise could have likely impaired the cross-bridge cycle efficiency by a higher extent in *VM* compared to *VL* and *RF*. Further studies are needed to explore these two possible mechanisms. Though no direct comparison with the literature can be made, our data suggest the need to distinguish the effects of exercise-induced fatigability in synergistic muscles, since they may respond differently to a fatiguing task.

In the contralateral non-exercising limb, the VA_MMG_ decreased similarly in all muscle heads, as observed in the MMGp-p_MVC_, MMG RMS, MMG MF, sEMG RMS, and sEMG RMS/M-wave, with smaller reductions than the exercising limb. Regarding VA, the crossover effect for VA_MMG_ may be ascribed to central mechanisms, since the contralateral non-exercising muscle was not involved in the fatiguing exercise (as also confirmed by the similar M-waves and potentiated force values in POST compared to PRE). Although this approach is novel in the literature, a previous study showed that the contralateral non-exercising stretch-induced effects in the MMG signal were related to the central but not the peripheral components (Cè et al. 2020a). This may also account for the lack of between-muscle difference observed in the contralateral non-exercising muscle, suggesting that the greater fatigue-induced decrease in VA_MMG_ in *VM* of the exercising limb could be due mainly by peripheral mechanisms.

The baseline measurements for VA and VA_MMG_ and the subsequent changes showed *moderate* correlations irrespective of the muscle in both the exercising and the contralateral non-exercising limb. VA and VA_MMG_ are the expression of voluntary activation mechanisms, including the motor drive, the Ca^2+^ transient, and the level of muscle activation (Orizio 1993; Orizio et al. 2003; Gobbo et al. 2006; de Haan et al. 2009). However, the previous studies have found that MMGp-p_SUP_ amplitude has a greater correlation with the level of fascicle shortening during contraction than with superimposed twitch amplitude (Ohta et al. 2007, 2009). Such a discrepancy between the two variables fits the equation for calculating VA_MMG_ and VA, and could explain the difference between the percentage of exercise-induced decrease in VA_MMG_ and VA reported here. Finally, the lack of correlation between VA_MMG_ and the sEMG variables is likely due to the different nature of the mechanisms of their occurrence; while VA_MMG_ is also affected by the excitation–contraction coupling and the muscle mechanical properties, the sEMG variables are influenced by the excitability of the motoneurons and the sarcolemma’s action potential transmission properties (Orizio 1993; Merletti et al. 2003; Cè et al. 2015).

The VA_MMG_ data resulted in *very high* inter-session reliability, accompanied by small SEM%. Concurrently, the low levels of MDC_95%_ observed for the VA_MMG_ highlight adequate sensitivity to detect exercise-induced variations in the muscle heads in both limbs. The observed reliability of VA is shared by a previous study (Cè et al. 2020a), whereas the reliability of VA_MMG_ is novel and cannot be compared. Moreover, the MMG signal crosstalk values spanned from 4 to 11%, in line or lower than the data reported in the previous studies (Beck et al. 2010; Islam et al. 2014; Ismail et al. 2021). These results suggest that the outcomes regarding VA_MMG_ behavior were minimally influenced by possible methodological biases derived from this new approach.

The present study has several limitations. First, since the between-muscle difference was not determined by any metabolic assessment, no deeper mechanistic explanation for the observed results can be given. Second, our observations are related to the muscles investigated and to the fatiguing task used here; other synergistic muscles or other types of exercise may yield different findings. Similarly, the present results refer to the present population and should not be generalized (e.g., in women). Moreover, as recently confirmed by a review (Dotan et al. 2021), there are several limitations related to the use of the twitch interpolation technique in assessing VA. The use of other approaches such as the transcranial magnetic stimulation could have provided a more accurate evaluation of VA. Finally, as reported in a previous investigation (Mira et al. 2017), the 2 min delay between the end of exercise and the post-fatigue assessments could have possibly introduced an underestimation of the decrease in VA, as central fatigue has been shown to recover quickly after exercise.

## Conclusions

The interpolated twitch technique does not allow to distinguish exercise-induced changes in VA for each individual superficial head of the quadriceps muscle; nonetheless, VA_MMG_ can be used to examine non-invasively the behavior of the synergistic superficial muscles. Given the high reliability and sensitivity of the VA_MMG_, as well as its responsiveness and correlation with VA, VA_MMG_ may be used alone or in conjunction with the force signal to investigate the effects of exercise-induced fatigability. This opens novel methodological perspectives to study the effects of fatigue.

## Supplementary Information

Below is the link to the electronic supplementary material.

Supplementary file1 (DOCX 58 KB)

## Data Availability

The data that support the findings of this study will be deposited in open access on the database Zenodo (md5:6e4ae62413890d903920adba94068a14).

## Abbreviations

sEMG: Surface electromyography
MF: Mean frequency
MMG: Mechanomyography
MMGp-p_MVC_: MMG peak-to peak during maximum voluntary contraction,
MMGp-p_POT_: Potentiated twitch MMG peak-to-peak at rest
MMGp-p_SUP_: Superimposed MMG peak-to-peak
MVC: Maximum voluntary contraction
POST: After
PRE: Before
RMS: Root mean square
RF: *rectus femoris*
VA: Voluntary activation
VA_MMG_: VA recorded by the MMG signal
VL: *vastus lateralis*
VM: *vastus medialis*
