# Supplementary material for: Determining voluntary activation in synergistic muscles: a novel mechanomyographic approach
Source: Eur J Appl Physiol. 2022 May 24;122(8):1897–913. doi: 10.1007/s00421-022-04966-8 (PMC9287262; doi:10.1007/s00421-022-04966-8)
Supplement: Supplementary file 1 — Supplementary file1 (DOCX 58 KB) [file 421_2022_4966_MOESM1_ESM.docx]

| **Mechanomyogram Root Mean Square (MMG RMS, mm⋅s^-2^), N = 25** | | | | | | |
| --- | --- | --- | --- | --- | --- | --- |
| Condition | *Limb* | *Muscle* | *PRE*  *[m (SD]* | *POST*  *[m (SD]* | *Cohen’s d*  *(CI_95%_)* | *P value* |
| Exercise ^c^ | Exercising* | *VL* | 0.793 (0.100) | 0.637 (0.082) | -1.71 (-2.02 to -1.39) | <0.001 |
|  |  | *VM*^†^ | 0.735 (0.103) | 0.550 (0.072) | -2.09 (-2.42 to -1.75) | <0.001 |
|  |  | *RF* | 0.777 (0.100) | 0.639 (0.105) | -1.35 (-1.65 to -1.05) | <0.001 |
|  | Contralateral | *VL* | 0.793 (0.106) | 0.746 (0.100) | -0.46 (-0.73 to -0.19) | <0.001 |
|  |  | *VM* | 0.737 (0.104) | 0.702 (0.101) | -0.34 (-0.61 to -0.07) | <0.001 |
|  |  | *RF* | 0.776 (0.100) | 0.739 (0.097) | -0.38 (-0.65 to -0.11) | <0.001 |
| Control | Exercising | *VL* | 0.794 (0.099) | 0.793 (0.101) | 0.00 (-0.27 to 0.27) | 0.960 |
|  |  | *VM* | 0.735 (0.101) | 0.736 (0.103) | 0.01 (-0.26 to 0.28) | 0.525 |
|  |  | *RF* | 0.776 (0.100) | 0.775 (0.101) | -0.02 (-0.28 to 0.25) | 0.330 |
|  | Contralateral | *VL* | 0.798 (0.099) | 0.793 (0.106) | -0.05 (-0.32 to 0.22) | 0.281 |
|  |  | *VM* | 0.737 (0.106) | 0.738 (0.106) | 0.01 (-0.26 to 0.28) | 0.279 |
|  |  | *RF* | 0.779 (0.101) | 0.777 (0.100) | -0.01 (-0.28 to 0.26) | 0.289 |
|  |  |  |  |  |  |  |
| **Mechanomyogram Mean Frequency (MMG MF, Hz), N = 25** | | | | | | |
| Condition | *Limb* | *Muscle* | *PRE*  *[m (SD]* | *POST*  *[m (SD]* | *Cohen’s d*  *(CI_95%_)* | *P value* |
| Exercise ^c^ | Exercising* | *VL* | 20.8 (3.0) | 16.9 (2.9) | -1.33 (-1.63 to -1.03) | <0.001 |
|  |  | *VM*^†^ | 20.7 (3.0) | 16.0 (2.7) | -1.63 (-1.94 to -1.32) | <0.001 |
|  |  | *RF* | 20.8 (2.9) | 17.4 (2.5) | -1.22 (-1.52 to -0.93) | <0.001 |
|  | Contralateral | *VL* | 20.8 (2.9) | 19.9 (2.7) | -0.32 (-0.59 to -0.05) | <0.001 |
|  |  | *VM* | 20.8 (2.9) | 19.7 (3.0) | -0.36 (-0.64 to -0.09) | <0.001 |
|  |  | *RF* | 20.7 (2.8) | 19.9 (2.8) | -0.29 (-0.56 to -0.02) | <0.001 |
| Control | Exercising | *VL* | 20.8 (3.0) | 20.8 (3.0) | 0.02 (-0.25 to 0.29) | 0.483 |
|  |  | *VM* | 20.6 (2.9) | 20.7 (2.9) | -0.02 (-0.29 to 0.25) | 0.522 |
|  |  | *RF* | 20.7 (2.9) | 20.6 (2.9) | 0.01 (-0.25 to 0.28) | 0.570 |
|  | Contralateral | *VL* | 20.8 (2.8) | 20.9 (2.9) | 0.04 (-0.22 to 0.31) | 0.245 |
|  |  | *VM* | 20.7 (2.9) | 20.8 (2.8) | 0.02 (-0.25 to 0.28) | 0.345 |
|  |  | *RF* | 20.7 (2.7) | 20.7 (2.7) | -0.03 (-0.30 to 0.24) | 0.310 |

**Table, Supplemental Digital Content 1.** Root mean square and mean frequency of the mechanomyographic signal of the three muscles in the exercising and contralateral non-exercising limb, before (PRE) and after (POST) exercise and control. The post-pre differences were calculated using Cohen’s *d* effect size (95% CI) and *P*-value.

c = *P* <0.05 vs Control

* = *P* <0.05 vs Contralateral

† = *P* <0.05 vs. *VL* and *RF*

**Table, Supplemental Digital Content 2.** Peak-to-peak of the mechanomyographic signal during maximum voluntary contraction (MMGp-p_MVC_), superimposed (MMGp-p_SUP_), or potentiated (MMGp-p_POT_) from the three muscles in the exercising and the contralateral non-exercising limb before (PRE) and after (POST) exercise or control. The post-pre differences were calculated using Cohen’s *d* effect size (95% CI) and *P*-values.

c = *P* <0.05 vs Control

* = *P* <0.05 vs Contralateral

† = *P* <0.05 vs. *VL* and *RF*

| **Mechanomyogram peak-to-peak during maximum voluntary contraction (MMGp-p_MVC_, mm⋅s^-2^), N = 25** | | | | | | |
| --- | --- | --- | --- | --- | --- | --- |
| Condition | *Limb* | *Muscle* | *PRE*  *[m (SD]* | *POST*  *[m (SD]* | *Cohen’s d*  *(CI_95%_)* | *P value* |
| Exercise^c^ | Exercising* | *VL* | 8.6 (2.1) | 6.7 (1.9) | -0.93 (-1.22 to -0.65) | <0.001 |
|  |  | *VM* † | 8.5 (2.2) | 6.3 (1.9) | -1.16 (-1.45 to -0.87) | <0.001 |
|  |  | *RF* | 8.6 (2.2) | 7.0 (1.9) | -0.78 (-1.06 to -0.50) | <0.001 |
|  | Contralateral | *VL* | 8.5 (2.1) | 8.0 (2.1) | -0.23 (-0.50 to -0.04) | <0.001 |
|  |  | *VM* | 8.7 (2.2) | 8.4 (2.1) | -0.15 (-0.42 to 0.02) | <0.001 |
|  |  | *RF* | 8.6 (2.2) | 8.2 (2.1) | -0.20 (-0.47 to -0.07) | <0.001 |
| Control | Exercising | *VL* | 8.5 (2.1) | 8.5 (2.2) | -0.01 (-0.28 to 0.26) | 0.719 |
|  |  | *VM* | 8.5 (2.2) | 8.5 (2.1) | 0.01 (-0.26 to 0.28) | 0.732 |
|  |  | *RF* | 8.5 (2.2) | 8.5 (2.2) | -0.01 (-0.28 to 0.25) | 0.556 |
|  | Contralateral | *VL* | 8.6 (1.9) | 8.5 (1.9) | -0.03 (-0.30 to 0.24) | 0.241 |
|  |  | *VM* | 8.7 (2.1) | 8.7 (2.1) | 0.00 (-0.27 to 0.27) | 0.874 |
|  |  | *RF* | 8.7 (2.1) | 8.7 (2.1) | 0.01 (-0.26 to 0.28) | 0.669 |
|  |  |  |  |  |  |  |
| **Mechanomyogram peak-to-peak superimposed (MMGp-p_SUP_, mm⋅s^-2^), N = 25** | | | | | | |
| Condition | *Limb* | *Muscle* | *PRE*  *[m (SD]* | *POST*  *[m (SD]* | *Cohen’s d (CI_95%_)* | *P value* |
| Exercise^c^ | Exercising* | *VL* | 0.95 (0.37) | 1.09 (0.40) | 0.37 (0.10 to 0.64) | <0.001 |
|  |  | *VM*† | 0.98 (0.36) | 1.38 (0.53) | 0.85 (0.57 to 1.13) | <0.001 |
|  |  | *RF* | 0.98 (0.36) | 1.22 (0.49) | 0.48 (0.20 to 0.75) | <0.001 |
|  | Contralateral | *VL* | 0.95 (0.36) | 1.16 (0.46) | 0.51 (0.24 to 0.78) | <0.001 |
|  |  | *VM* | 1.00 (0.35) | 1.18 (0.40) | 0.50 (0.23 to 0.77) | <0.001 |
|  |  | *RF* | 0.98 (0.34) | 1.15 (0.35) | 0.50 (0.23 to 0.75) | <0.001 |
| Control | Exercising | *VL* | 0.95 (0.36) | 0.94 (0.36) | -0.03 (-0.30 to 0.24) | 0.251 |
|  |  | *VM* | 0.98 (0.36) | 0.99 (0.35) | 0.04 (-0.23 to 0.31) | 0.626 |
|  |  | *RF* | 0.97 (0.34) | 0.97 (0.35) | 0.01 (-0.26 to 0.28) | 0.787 |
|  | Contralateral | *VL* | 0.95 (0.35) | 0.94 (0.34) | -0.01 (-0.28 to 0.27) | 0.560 |
|  |  | *VM* | 0.99 (0.34) | 1.00 (0.34) | 0.04 (-0.21 to 0.29) | 0.375 |
|  |  | *RF* | 0.98 (0.34) | 0.98 (0.33) | 0.01 (-0.23 to 0.22) | 0.513 |
|  |  |  |  |  |  |  |
| **Mechanomyogram peak-to-peak during single twitch at rest (MMGp-p_POT_, mm⋅s^-2^), N = 25** | | | | | | |
| Condition | *Limb* | *Muscle* | *PRE*  *[m (SD]* | *POST*  *[m (SD]* | *Cohen’s d (CI_95%_)* | *P value* |
| Exercise^c^ | Exercising* | *VL* | 7.8 (1.9) | 6.1 (1.7) | -0.93 (-1.21 to -0.65) | <0.001 |
|  |  | *VM*† | 7.9 (2.0) | 5.8 (1.7) | -1.13 (-1.42 to 0.84) | <0.001 |
|  |  | *RF* | 7.9 (2.0) | 6.4 (1.7) | -0.79 (-1.07 to -0.51) | <0.001 |
|  | Contralateral | *VL* | 7.8 (1.9) | 7.8 (1.9) | 0.00 (-0.27 to 0.27) | 0.569 |
|  |  | *VM* | 8.0 (1.9) | 7.9 (1.9) | 0.01 (-0.26 to 0.28) | 0.588 |
|  |  | *RF* | 7.9 (1.9) | 7.8 (2.1) | -0.01 (-0.28 to 0.26) | 0.459 |
| Control | Exercising | *VL* | 7.8 (1.9) | 7.8 (1.9) | -0.01 (-0.28 to 0.26) | 0.345 |
|  |  | *VM* | 7.8 (2.0) | 7.9 (1.9) | 0.01 (-0.26 to 0.28) | 0.720 |
|  |  | *RF* | 7.8 (2.0) | 7.8 (2.0) | -0.01 (-0.28 to 0.26) | 0.531 |
|  | Contralateral | *VL* | 7.9 (1.8) | 7.9 (1.8) | -0.03 (-0.30 to 0.24) | 0.300 |
|  |  | *VM* | 8.0 (1.9) | 8.0 (1.8) | 0.00 (-0.27 to 0.27) | 0.758 |
|  |  | *RF* | 8.0 (1.9) | 8.0 (1.9) | 0.01 (-0.26 to 0.28) | 0.461 |

**Table, Supplemental Digital Content 3.** Root mean square of the surface electromyographic signal (sEMG RMS), M-wave, and sEMG RMS/M-wave ratio of the three muscles in the exercising and contralateral non-exercising limb, before (PRE) and after (POST) exercise and control. The post-pre differences were calculated using Cohen’s *d* effect size (95% CI) and *P*-value.

c = *P* <0.05 vs Control

* = *P* <0.05 vs Contralateral

† = *P* <0.05 vs. *VL* and *RF*

|  | | **Surface Electromyogram Root Mean Square (sEMG RMS, mV), N = 25** | | | | | | |
| --- | --- | --- | --- | --- | --- | --- | --- | --- |
| Condition | *Limb* | | *Muscle* | *PRE*  *[m (SD]* |  | *POST*  *[m (SD]* | *Cohen’s d*  *(CI_95%_)* | *P value* |
| Exercise^c^ | Exercising* | | *VL* | 0.841 (0.033) |  | 0.645 (0.150) | -0.61 (-1.18 to -0.04) | <0.001 |
|  |  |  | *VM* † | 0.861 (0.052) |  | 0.538 (0.120) | -0.97 (-1.55 to -0.38) | <0.001 |
|  |  |  | *RF* | 0.731 (0.087) |  | 0.568 (0.150) | -0.57 (-1.14 to -0.00) | <0.001 |
|  | Contralateral | | *VL* | 0.870 (0.029) |  | 0.702 (0.024) | -0.48 (-1.04 to 0.08) | <0.001 |
|  |  |  | *VM* | 0.891 (0.064) |  | 0.716 (0.078) | -0.50 (-1.07 to -0.06) | <0.001 |
|  |  |  | *RF* | 0.762 (0.113) |  | 0.626 (0.130) | -0.34 (-0.90 to 0.21) | <0.001 |
| Control | Exercising* | | *VL* | 0.836 (0.058) |  | 0.837 (0.040) | 0.03 (-0.52 to 0.59) | 0.787 |
|  |  |  | *VM* | 0.856 (0.060) |  | 0.868 (0.115) | 0.13 (-0.42 to 0.69) | 0.557 |
|  |  |  | *RF* | 0.729 (0.088) |  | 0.739 (0.134) | 0.08 (-0.47 to 0.64) | 0.606 |
|  | Contralateral | | *VL* | 0.864 (0.058) |  | 0.866 (0.038) | 0.04 (-0.52 to 0.59) | 0.787 |
|  |  |  | *VM* | 0.886 (0.071) |  | 0.889 (0.070) | 0.04 (-0.52 to 0.59) | 0.815 |
|  |  |  | *RF* | 0.760 (0.113) |  | 0.757 (0.090) | -0.03 (-0.58 to 0.52) | 0.821 |
|  |  | |  |  |  |  |  |  |
|  | | ***Surface Electromyogram M-wave* (*mV*), N = 25** | | | | | | |
| Condition | *Limb* | | *Muscle* | *PRE*  *[m (SD]* |  | *POST*  *[m (SD]* | *Cohen’s d (CI_95%_)* | *P value* |
| Exercise^c^ | Exercising* | | *VL* | 3.95 (1.51) |  | 3.58 (1.34) | -0.25 (-0.81 to 0.30) | <0.001 |
|  |  |  | *VM* | 3.13 (1.17) |  | 2.88 (1.14) | -0.21 (-0.77 to 0.34) | <0.001 |
|  |  |  | *RF* | 3.05 (1.08) |  | 2.89 (1.04) | -0.15 (-0.71 to 0.40) | <0.001 |
|  | Contralateral | | *VL* | 4.02 (1.80) |  | 4.05 (1.81) | 0.02 (-0.54 to 0.57) | 0.194 |
|  |  |  | *VM* | 3.13 (1.16) |  | 3.14 (1.18) | 0.01 (-0.54 to 0.57) | 0.587 |
|  |  |  | *RF* | 3.05 (1.15) |  | 3.05 (1.09) | 0.01 (-0.55 to 0.56) | 0.760 |
| Control | Exercising | | *VL* | 3.91 (1.47) |  | 3.91 (1.53) | 0.00 (-0.56 to 0.55) | 0.953 |
|  |  |  | *VM* | 3.09 (1.08) |  | 3.11 (1.12) | 0.01 (-0.54 to 0.57) | 0.451 |
|  |  |  | *RF* | 3.05 (1.17) |  | 3.08 (1.15) | 0.05 (-0.55 to 0.55) | 0.809 |
|  | Contralateral | | *VL* | 4.00 (1.82) |  | 4.08 (1.97) | 0.04 (-0.51 to 0.60) | 0.195 |
|  |  |  | *VM* | 3.12 (1.18) |  | 3.16 (1.18) | 0.03 (-0.52 to 0.59) | 0.475 |
|  |  |  | *RF* | 3.04 (1.19) |  | 3.07 (1.17) | 0.04 (-0.52 to 0.59) | 0.451 |
|  |  |  |  |  |  |  |  |  |
|  | | ***sEMG RMS/M-wave*, N = 25** | | | | | | |
| Condition | *Limb* | | *Muscle* | *PRE*  *[m (SD]* |  | *POST*  *[m (SD]* | *Cohen’s d (CI_95%_)* | *P value* |
| Exercise^c^ | Exercising* | | *VL* | 0.250 (0.108) |  | 0.210 (0.098) | -0.38 (-1.94 to 0.18) | 0.002 |
|  |  |  | *VM*† | 0.317 (0.130) |  | 0.218 (0.113) | -0.80 (-1.38 to -0.22) | <0.001 |
|  |  |  | *RF* | 0.253 (0.074) |  | 0.205 (0.067) | -0.68 (-1.25 to -0.11) | 0.002 |
|  | Contralateral | | *VL* | 0.262 (0.119) |  | 0.210 (0.094) | -0.48 (-1.04 to 0.08) | <0.001 |
|  |  |  | *VM* | 0.326 (0.133) |  | 0.263 (0.112) | -0.50 (-1.07 to 0.06) | <0.001 |
|  |  |  | *RF* | 0.264 (0.082) |  | 0.219 (0.083) | -0.54 (-1-10 to 0.03) | <0.001 |
| Control | Exercising | | *VL* | 0.249 (0.103) |  | 0.253 (0.110) | 0.04 (-0.52 to 0.59) | 0.533 |
|  |  |  | *VM* | 0.314 (0.124) |  | 0.322 (0.147) | 0.05 (-0.50 to 0.61) | 0.446 |
|  |  |  | *RF* | 0.252 (0.071) |  | 0.257 (0.089) | 0.05 (-0.61 to 0.50) | 0.516 |
|  | Contralateral | | *VL* | 0.263 (0.118) |  | 0.261 (0.120) | -0.01 (-0.57 to 0.54) | 0.662 |
|  |  |  | *VM* | 0.325 (0.132) |  | 0.322 (0.129) | -0.02 (-0.58 to 0.53) | 0.661 |
|  |  |  | *RF* | 0.260 (0.073) |  | 0.265 (0.082) | -0.05 (-0.61 to 0.50) | 0.275 |
